# Clinical Implications of Oscillatory Lung Function during Methacholine Bronchoprovocation Testing of Preschool Children

**DOI:** 10.1155/2017/9460190

**Published:** 2017-06-27

**Authors:** Sun Hee Choi, Youn Ho Sheen, Mi Ae Kim, Ji Hyeon Baek, Hey Sung Baek, Seung Jin Lee, Jung Won Yoon, Yeong Ho Rha, Man Yong Han

**Affiliations:** ^1^Department of Pediatrics, Kyung Hee University School of Medicine, Seoul, Republic of Korea; ^2^Department of Pediatrics, CHA University School of Medicine, Seoul, Republic of Korea; ^3^Department of Medicines, CHA University School of Medicine, Seongnam, Republic of Korea; ^4^Department of Pediatrics, Dongtan Sacred Heart Hospital, College of Medicine, Hallym University, Hwaseong, Republic of Korea; ^5^Department of Pediatrics, Kangdong Sacred Heart Hospital, Hallym University, Seoul, Republic of Korea; ^6^Department of Pediatrics, Myongji Hospital, Seonam University College of Medicine, Goyang, Republic of Korea

## Abstract

**Objective:**

To investigate the repeatability and safety of measuring impulse oscillation system (IOS) parameters and the point of wheezing during bronchoprovocation testing of preschool children.

**Methods:**

Two sets of methacholine challenge were conducted in 36 asthma children. The test was discontinued if there was a significant change in reactance (Xrs5) and resistance (Rrs5) at 5 Hz (Condition 1) or respiratory distress due to airway obstruction (Condition 2). The repeatability of PC_80__Xrs5, PC_30__Rrs5, and wheezing (PCw) was assessed. The changes in *Z*-scores and SD-indexes from prebaseline (before testing) to postbaseline (after bronchodilator) were determined.

**Results:**

For PC_30__Rrs5, PC_80__Xrs5, and PCw for subjects, PC_80__Xrs5 showed the highest repeatability. Fifteen of 70 tests met Condition 2. The changes from pre- and postbaseline values varied significantly for Rrs5 and Xrs5. Excluding subjects with *Z*-scores higher than 2SD, we were able to detect 97.1% of bronchial hyperresponsiveness during methacholine challenge based on the change in Rrs5 or Xrs5. A change in IOS parameters was associated with wheezing at all frequencies.

**Conclusion:**

Xrs5 and Rrs5 have repeatability comparable with FEV1, and Xrs5 is more reliable than Rrs5. Clinicians can safely perform a challenge test by measuring the changes in Rrs5, Xrs5, and *Z*-scores from the prebaseline values.

## 1. Introduction

Airway responsiveness to nonspecific stimuli, such as methacholine, is an important tool for diagnosis of asthma and monitoring the responses to asthma therapies in children and adults [1, 2]. Forced oscillation techniques are considered an alternative to spirometry for assessing the lung function of preschool children with bronchial hyperresponsiveness (BHR) [3], and various impulse oscillation system (IOS) parameters can be used for diagnosis of BHR [1, 4–9]. However, before the IOS method can be used in routine clinical practice, further research is needed to determine the repeatability of reactance and resistance measurements at all frequencies. Previous studies of a small number of children [2] and two adult populations [10, 11] have demonstrated the repeatability of these measurements, but study of a larger sample of children using different IOS parameters at different frequencies is warranted. In addition, although the IOS can detect early stage BHR, it may not be able to identify subjects who are about to develop airway obstruction [1, 6, 12, 13] because IOS parameters depend on the state of the patient. Thus, if a patient has a partial small airway dysfunction before the test, it is likely that the IOS will yield a low baseline value [10, 14, 15], and this may underestimate subsequent airway obstruction.

The present study of preschool children with asthma had two main objectives. First, we aimed to assess the repeatability of IOS parameters at all frequencies. Second, we aimed to determine the magnitude of changes in different IOS parameters and their deviations to guide clinicians on when they should stop the methacholine challenge test.

## 2. Methods

### 2.1. Subjects

We enrolled children with asthma who presented to the Department of Pediatric Pulmonology, CHA University, Bundang Hospital, Seongnam, Korea (age range: 3–6 years), from March through August 2010. All subjects were diagnosed with asthma according to the 2007 Expert Panel Report 3. All children were asked to abstain from short- or long-acting bronchodilators for at least 48 h or a leukotriene modifier at least 24 h prior to the test [16]. This study was approved by the institutional review board of the CHA Bundang Medical Center, CHA University School of Medicine (2010-008), and written informed consent was obtained from the participants' parents upon enrollment.

### 2.2. Study Design

This was a prospective observational study based on data collected from children at two hospital visits at the same time of the day with at least 3 days' interval. After obtaining written informed consent, the patients underwent the methacholine challenge test using IOS.

### 2.3. Pulmonary Function and the Methacholine Challenge Test

The methacholine challenge test was performed according to published guidelines [17], with a doubling of the concentration of the methacholine solution (0.25, 0.5, 1, 2, 4, 8, and 16 mg/mL methacholine) in normal saline. Methacholine chloride aerosols were generated by calibrated DeVillbiss 646 nebulizers (pretest mean output 0.26 ± 0.02 mg/min and posttest 0.23 ± 0.03 mg/min) utilizing tidal breathing through a mouthpiece for 2 minutes. Chest auscultation and oxygen saturation monitoring were performed during the first 30 s after the end of each methacholine dose. The bronchoprovocation test was continued as long as the child was cooperative and was stopped if any of 2 predetermined conditions were met. Condition 1 was defined as a 30% or more change in Rrs5 (PC_30__Rrs5) and an 80% or more change in Xrs5 (PC_80__Xrs5) from the baseline values [2, 4]. Condition 2 was defined as difficulty in breathing or a change in oxygen saturation of at least 5% from baseline (desaturation). If the inhaled methacholine concentration reached 16 mg/mL, the test was discontinued. A positive response to the methacholine challenge test was defined by the presence of any of the following: (i) wheezing based on auscultation of the chest and trachea (double-checked by two pediatricians, Dr. Han MY and Dr. Choi SH); (ii) a 30% or more increase in the resistance value at 5 Hz; or (iii) an 80% or more increase in the reactance value at 5 Hz.

IOS measurements (MasterScreen IOS, Jaeger, Germany) were performed according to American Thoracic Society (ATS)/European Respiratory Society (ERS) guidelines [4, 12]. For quality control, the physicians confirmed their results by visual monitoring and coherence and calculation of the coefficients of variation (CVs) [1, 3, 12]. After the final step of the methacholine test, subjects were given 2.5 mg of salbutamol.

We calculated the changes from before the methacholine challenge (prebaseline) to after the challenge (postbaseline, after the test and salbutamol administration). The prebaseline % was defined as the percent change of a parameter at different concentrations of methacholine from the prebaseline value, and the postbaseline % as the percent change of a postbaseline parameter. The SD-index and* Z*-score were calculated at each methacholine dose to determine the extent of deviation to be used for further comparisons [18]. The SD-index was obtained by dividing the change from baseline values by the within-subject SD (SDw), which was calculated by dividing the difference between the mean values of the first and second measurements by the square root of 2 [14].* Z-*scores were calculated as described by Frei et al. [19]. Values are expressed as PC_SDi*n*__*Q*, where *Q* refers to the parameter, and *n* refers to the deviation. Airway resistance was indicated by a *Z*-score of at least 2, or an SD-index of at least 3 [13].

### 2.4. Data Analysis

Data are presented as means and standard deviations, unless otherwise indicated. Student's* t*-test was used to compare paired data (prebaseline versus postbaseline values) and the independent samples in Condition 1 and 2. The agreement of positive response ratios between Rrs5 and Xrs5 (based on prebaseline and postbaseline values) was analyzed using the kappa coefficient (*κ*). For each of the methods used to determine BHR, the within-subject standard deviation (SDw), coefficient variation (CV), and the intraclass correlation coefficient (ICC) were estimated to compare the repeatability of PC_%_ [20]. The PC_%_ was calculated for each method; PC_30__Rrs5 and PC_80__Xrs5 were calculated from linear interpolation of the log_10_(dose)-response curves. The PC_%_ was calculated as 0.01 mg/dL for zero dose and 32 mg/dL if a sufficient change was not achieved after the last dose. PCw was defined as the concentration at which wheezing or desaturation (more than 5% from baseline) without wheezing developed.

The minimal sample size of the current study was 32, based on the primary outcome, mean, and SD of Rrs5, as described by Klug and Bisgaard [2]. This would allow discrimination of results with 90% power at the 5% significance level. The repeatability of PC_%_ was compared by an *F*-test. *p* values less than 0.05 were considered statistically significant.

## 3. Results

Thirty-six children participated in the study and had at least one visit for IOS testing (Table 1). Two children (both 2 years old; one boy and one girl) did not complete the second challenge test and were not included in the repeatability analysis. There were no significant differences in baseline measurements of the two tests (Table 2).

### 3.1. Repeatability of BHR in Each MCT

**Figure 1:**
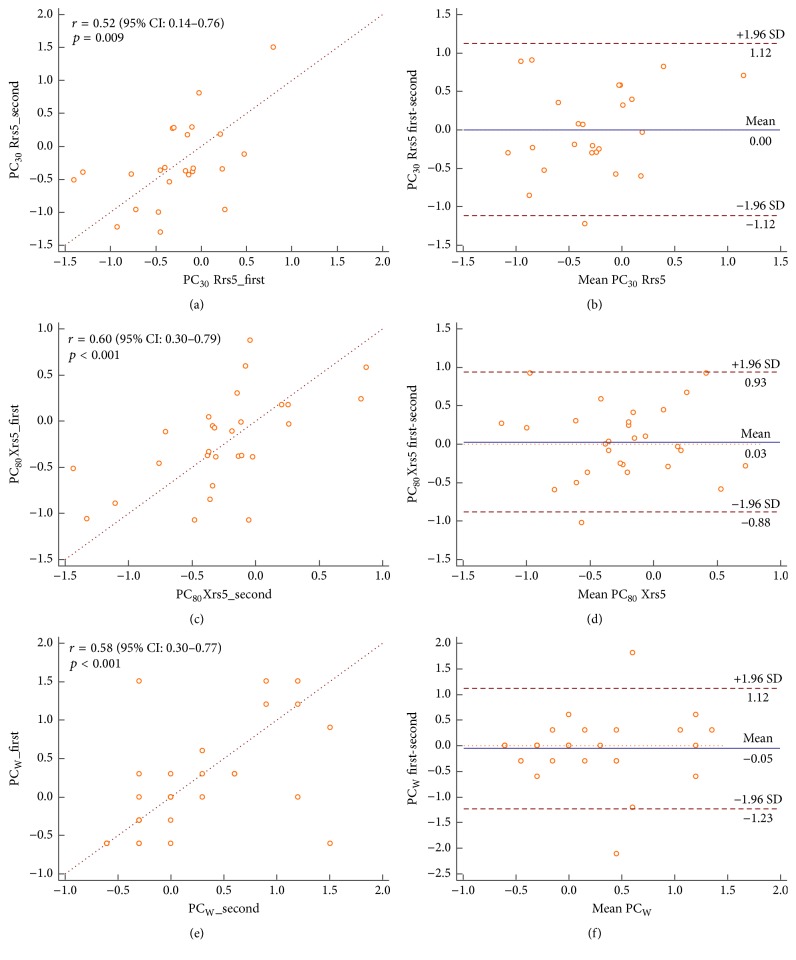
Reproducibility of PC_30_Rrs5, PC_80_Xrs5, and PC_W_ for two measurements indicated by Spearman correlation and Bland-Altman plot. PC_80_Xrs5 shows the highest repeatability among PC_30_Rrs5 (a, b), PC_80_Xrs5 (c, d), and PC_W_ (e, f). The data is log-transformed.

We measured the repeatability of bronchial responsiveness in resistance and reactance at 5 Hz and auscultation. The doubling concentration and the mean difference of the PC values were significantly correlated with each other, whereas the PC_30_Rrs5, PC_80_Xrs5, and PCw for two measurements were moderately correlated with one another. Coefficient of repeatability (CR) for those parameter was 2.56, 1.54, and 1.51 and for ICC, 0.68 (0.29–0.86), 0.76 (0.48–0.89), and 0.74 (0.48–0.89), respectively. Evaluation of repeatability indicated that PC_80_Xrs5 was more reproducible than PC_30_Rrs5 and PCw in all cases (Figures 1(a)–1(f)). Rrs5 (which is based on the *Z*-score) yielded a doubling concentration of 0.78 (95% CI, 0.49–1.07) and a SD-index 3 of 0.83 (95%, CI 0.56–1.08), thus showing good repeatability. The ICC was greater than 0.6 for all methods (Table 3).

### 3.2. Comparison of IOS Parameters between Children with and without Clinical Signs and Symptoms of Respiratory Distress for End-Point during Provocation Test: Safety of Challenge Testing

**Figure 2:**
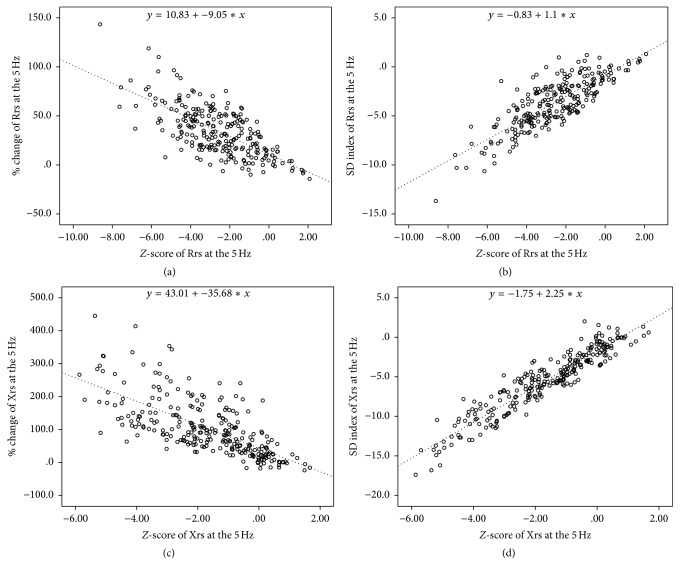
Correlation of* Z*-scores with relative changes of reactance and resistance and SD-indexes of reactance and resistance based on 245 measurements at 5 Hz. At* Z*-score resistance of 2, relative change was 28.93% (a) and the SD-index was 3.03 (b). At* Z*-score reactance of 2, relative change was 114.37% (c), and the SD-index was 6.25 (d).

**Figure 3:**
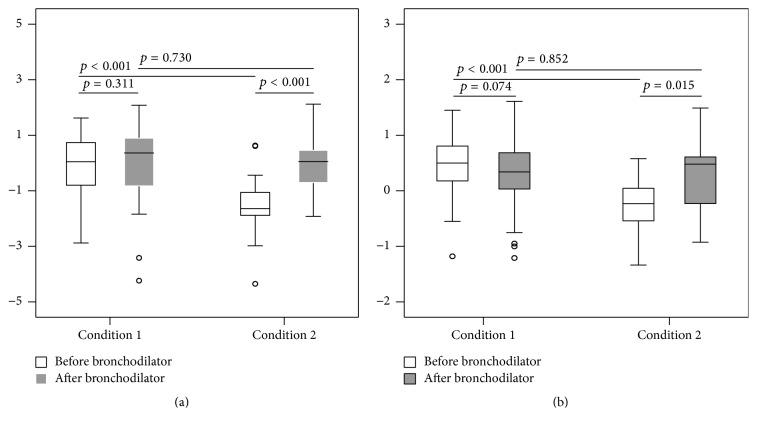
Prebaseline and postbaseline values under different conditions of stopping methacholine challenge test. Condition 1 (*n* = 55) was applied when the test was stopped because changes in resistance and reactance reached a certain point; Condition 2 (*n* = 15) was applied when the test was stopped due to signs of respiratory distress. Prebaseline values were significantly different between Conditions 1 and 2 with regard to resistance (*p* < 0.001) (a) and reactance (*p* < 0.001) (b). Pre- and postbaseline values in Condition 2 were significantly different with regard to resistance (*p* < 0.001) (a) and reactance (*p* = 0.015) (b).

We performed 70 challenge tests (36 children received the first test, and 34 received the second test) and used 245 lung function measurements for analysis (Figures 2(a)–2(d)). The baseline Rrs5 and Xrs5 *Z*-scores have a significant correlation with each change (%) and SD-index during provocation. We discontinued 55 of the 70 lung function tests (79%) because of Condition 1 and 15 tests (21%) because of Condition 2 (Figures 3(a) and 3(b)). In the Condition 1 group, there was no significant difference in the pre- and postbaseline values of Rrs5 (*p* = 0.311) and Xrs5 (*p* = 0.074), but there were significant differences of these parameters in the Condition 2 group (*p* < 0.001 for Rrs and *p* = 0.015 for Xrs). In addition, the two groups differed in their prebaseline values of Rrs5 (*p* < 0.001) and Xrs5 (*p* < 0.001), but not in their postbaseline values (*p* = 0.730 for Rrs5 and *p* = 0.820 for Xrs5).

The positive response rate before airway obstruction was 84.3% (*n* = 59) for Rrs5 according to the prebaseline value and 87.1% (*n* = 61) for Xrs5, showing modest agreement (*κ* = 0.418). However, the agreement decreased if analyzed according to postbaseline value (*κ* = 0.178). Although there were differences between these groups with regard to absolute changes, relative changes, and SD-indexes of Rrs and Xrs5 (parameters highly dependent on baseline values), there were no differences in the *Z*-score, a parameter that reflects absolute lung function changes (*p* = 0.336 for Rrs5, *p* = 0.779 for Xrs5) (Table 4). Sixty-eight tests were terminated based on changes in prebaseline values of Rrs5 (84.3%) or Xrs5 (87.1%) or prebaseline *Z*-scores, leading to an overall detection rate of 97.1%.

### 3.3. Wheezing and IOS Parameters

**Figure 4:**
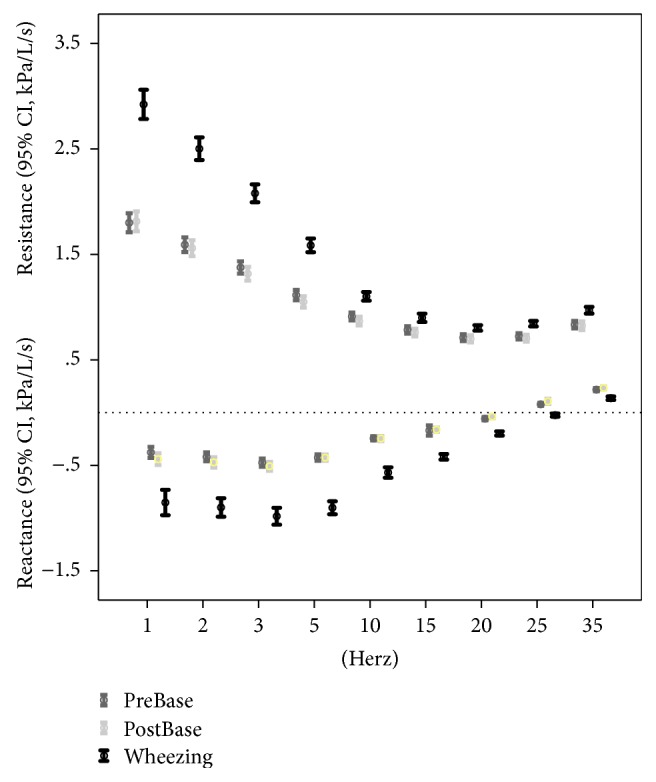
Resistance and reactance values at the wheezing point at all frequencies (1–35 Hz). There were significant differences between low (<3 Hz, small airway) and high (>25 Hz, large airway) frequencies.

**Figure 5:**
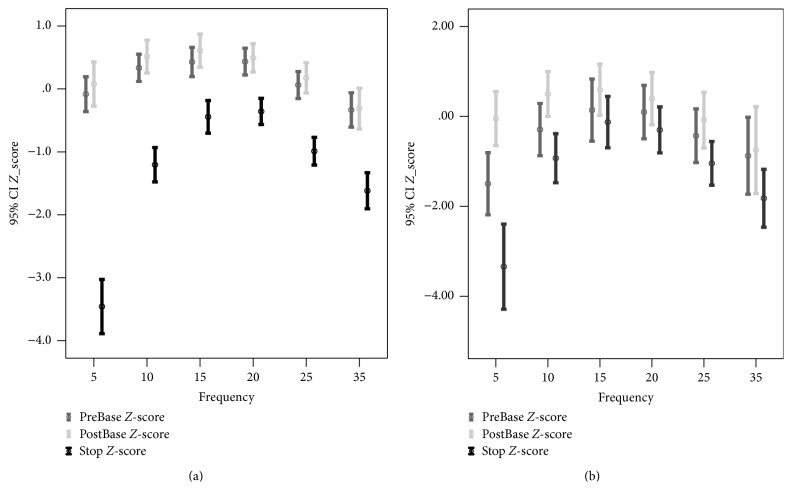
*Z*-scores of Rrs5 as a function of frequency at which wheezing developed. Condition 1 (*n* = 55) was applied when the test was stopped because changes in resistance and reactance reached a certain point (a); Condition 2 (*n* = 15) was applied when the test was stopped due to signs of respiratory distress (b). There were no significant differences in* Z*-scores in Conditions 1 and 2 (−3.46 ± 1.59 versus −3.34 ± 1.71, *p* = 0.808).

The resistance and reactance values changed at the point of wheezing at all frequencies and were particularly large at low frequencies (<5 Hz) reflecting a small airway dysfunction (Figure 4). The changes from prebaseline to the wheezing point were 45.7% (95% CI, 39.1–52.3) for Rrs5 and 124.0% (95% CI, 103.8–144.1) for Xrs5. The *Z*-scores at which wheezing developed at the first prebaseline *Z*-score for Rrs5 were −3.46 (95% CI, −0.91 to 1.14) and −3.34 (95% CI, −0.83 to 1.06), with no significant difference between the two groups (Figures 5(a) and 5(b)).

## 4. Discussion

The repeatability of reactance by IOS was comparable to that of FEV1 by spirometry and better than that of resistance by IOS. The repeatability was similar at all frequencies for all IOS parameters. Rrs and Xrs changed significantly from the prebaseline values prior to the development of respiratory distress in 84% of tests (Rrs) and 87% of tests (Xrs). The early detection rate increased if the test was terminated when either condition was met. The baseline Rrs5 and Xrs5 *Z*-scores showed a constant correlation formula with each change amount (%) and SD-index during provocation. We were able to predict the methacholine concentration in each subject at which he/she will likely develop wheezing or signs and symptoms of respiratory distress and thus to stop the test. The risk of a patient developing severe bronchospasm was reduced if the *Z*-score and the relative changes of Rrs5 or Xrs5 were used to determine airway obstruction. At the point of wheezing, the changes in resistance and reactance were 45.7% and 124.0%, respectively. These changes occurred at all frequencies (1–35 Hz) at which wheezing developed, indicating that it occurred simultaneously in all parts of the airways.

It is well known that repeated measurements with PC_20__FEV1 fall within 1.5 to 1.6 of the doubling dose [21, 22]. Inman et al. [21] showed that the difference between the two measurements is less than 1 doubling concentration in 95% of subjects. Furthermore, some studies that used a 24-h interval have reported a doubling dose as low as 0.8 [23]. In the IOS, a previous study of 16 preschool children indicated that repeated measurements at PC_80__Xrs5 and PC_30__Rrs5 were within 1.3 and 2.5 of the doubling doses, respectively [2]. A study of adults indicated that the doubling dose of PC_35__Rrs with a histamine challenge test was 1.11 [10], and the doubling dose of PC_40__Rrs6 was 2.7 [11]. Our study yielded lower doubling doses and we observed good repeatability at all frequencies and in all parameters. Thus, we conclude that the resistance and reactance parameters of IOS may be useful in clinical settings.

Interestingly, our study and other studies [2] have reported greater repeatability of reactance than resistance. This difference may be attributed to the glottic aperture, the most important site for controlling airflow through the upper airways [24], because it can affect the repeatability of resistance measurements [25]. The ICC in our study was comparable to that of previous studies [10, 21–23], and such values were similar in all parameters at all frequencies.

Baseline lung function by spirometry [26] and the IOS [27] are the major determinants for measuring BHR. It is well known that the IOS parameters, particularly Rrs, are more sensitive to changes in airway obstruction than other lung function tests [10, 14, 15, 28]. This may lead to higher fluctuations in the baseline values. Therefore, the calculated relative change of the IOS parameters could misrepresent the actual extent of airway obstruction, because baseline values of pre- and post-IOS often differ significantly [4, 7, 29]. This phenomenon does not occur in spirometry. Peták et al. suggested that this could be caused by the use of a beta-2 agonist [7]. However, this could also be due to higher levels of airway resistance in children with asthma at the time of the prebaseline measurements. The presence of a significantly lower prebaseline *Z*-score in Condition 2 than in Condition 1, but comparable postbaseline values, suggests that partial airway obstruction may have already been present at the time of the prebaseline measurements in the Condition 2 group and that this resolved following salbutamol treatment. This interpretation is supported by the presence of significant differences in absolute changes, relative changes, and SD-index values of patients with changes in resistance and those with no changes, but no difference in *Z*-scores between these two groups.

In the present study, we showed that the risk of a patient experiencing bronchospasm could be reduced by considering relative changes of Rrs5 or Xrs5; however, we were unable to find signs or symptoms of airway distress in 5 children using these criteria. The children who showed clinical signs and symptoms of respiratory distress but negative test results had significantly lower *Z*-scores. Thus, eliminating patients with outlier prebaseline *Z*-scores or discontinuing tests based on *Z*-scores may improve safety. We speculate that it would be appropriate to interpret the two tests independently, because the agreement between Xrs5 and Rrs5 was relatively low. Thus, we were able to increase the detection rate by considering the *Z*-score and relative changes in Xrs5 and Rrs5.

A strength of our study is the large number of patients, which increased the statistical power of our results. Also, calculating the *Z*-scores and SD-indexes for changes at all frequencies allowed comparisons with values measured at baseline. A limitation of our study is that we skipped the first three low doses of the methacholine challenge test. Another limitation is that we used a nebulizer kit that had a higher output than recommended by the ATS. Although Avital et al. [30] reported that a higher-output nebulizer may result in a different site of aerosol deposition, such high-output nebulizers are used in actual clinical settings, and the high repeatability of such a device at all frequencies of all IOS parameters should be recognized.

## 5. Conclusions 

We found that the repeatability of IOS parameters at all frequencies was comparable to that when using spirometry for the methacholine challenge test and that reactance had better repeatability than resistance. Using change of resistance and reactance, and comparison of those values with the prebaseline *Z*-score, allowed safe administration of methacholine challenge test without provoking airway obstruction. Changes in resistance occurred in the entire airways at the onset of wheezing.

## Figures and Tables

**Table 1:** General characteristics of 36 asthma patients.

Characteristic	Mean	% or 95% CI
Sex, male	15	42
Age, years	4.3	4.0–4.6
Height, m	1.08	1.05–1.10
BMI, kg/m^2^	15.7	15.1–16.3
Baseline SpO_2_, %	99	98.8–99.5
Respiratory rate, breaths/min	15	14.2–15.3
Test interval, days	9.7	7.4–12.1
Asthma duration, months	20.0	15.4–24.5
Asthma medication		
Step 1	4	11.1
Step 2	17	47.2
Step 3	8	22.2
Step 4	7	19.4

CI, confidence interval; BMI, body mass index; asthma medication step indicates the medication used prior to first IOS measurements, according to the 2007 Expert Panel Report 3.

**Table 2:** Baseline measurements of lung function at challenge test 1 and challenge test 2.

	1st set	1st set CV	2nd set	2st set CV	SDw	CV (%)	ICC
Rrs5 (kPa/L/sec)	1.11 ± 0.24	8.04 ± 11.9	1.10 ± 0.18	7.68 ± 4.0	0.37	4.64	0.88 (0.76–0.94)
Rrs10 (kPa/L/sec)	0.90 ± 0.17	5.33 ± 2.70	0.90 ± 0.13	5.88 ± 4.05	0.31	4.29	0.87 (0.74–0.94)
Xrs5 (kPa/L/sec)	−0.43 ± 0.12	10.4 ± 5.74	−0.41 ± 0.10	14.0 ± 7.74	0.29	5.78	0.81 (0.61–0.91)
Xrs10 (kPa/L/sec)	−0.23 ± 0.06	11.7 ± 6.01	−0.25 ± 0.09	14.4 ± 8.44	0.31	10.35	0.73 (0.46–0.87)
AX (kPa/L)	3.62 ± 1.36	11.2 ± 6.70	3.28 ± 1.28	14.4 ± 10.08	0.98	11.21	0.85 (0.71–0.93)
Rf (Hz)	22.0 ± 2.0	4.90 ± 2.81	21.6 ± 2.2	4.64 ± 4.47	1.37	3.36	0.76 (0.52–0.88)

CV, coefficient of variation; SDw, within-subject SD (SD of the mean difference between the 1st and 2nd sets divided by √2); CV (%), CV between the first and second sets; ICC, intraclass correlation coefficient (between-subject variance divided by the total variance).

**Table 3:** Repeatability of bronchial responsiveness in resistance and reactance at 5 Hz with impulse oscillation system and wheezing.

	Resistance at 5 Hz		Reactance at 5 Hz	
	PC_*Z*s2_	PC_SDi3_	PC_*Z*s2_	PC_SDi3_
Δ Doubling concentration (95% CI)	0.78 (0.49–1.07)	0.83 (0.56–1.08)	0.87 (0.63–1.11)	0.81 (0.57–1.05)
Within-subject SD	0.75	0.73	0.73	0.75
ICC (95% CI)	0.78 (0.45–0.89)	0.71 (0.35–0.87)	0.68 (0.28–0.86)	0.83 (0.65–0.91)
Coefficient of repeatability	1.43	1.46	1.51	1.45

Mean difference (SD) between the number of doubling concentrations of methacholine required to achieve PC at challenge tests 1 and 2. PC_*Z*S2_, provocative concentration at which the *Z*-score is 2; PC_SDi3_, provocative concentration at which the SD-index is 3; CI, confidence interval; ICC, intraclass correlation coefficient.

**Table 4:** IOS values according to the results of Rrs5 and Xrs5 prior to the development of airway obstruction.

	Rrs5			Xrs5		
	Positive (*n* = 59)	Negative (*n* = 11)	*p* value	Positive (*n* = 61)	Negative (*n* = 9)	*p *Value
Baseline value (SD)	1.07 (0.19)	1.34 (0.21)	<0.001	−0.41 (0.11)	0.56 (0.11)	<0.001
Change of *Z*-score (SD)	−0.13 (1.04)	−1.75 (1.24)	<0.001	0.41 (0.55)	−0.37 (0.49)	<0.001
Stop Abs change value (SD)	0.51 (0.16)	0.19 (0.10)	<0.001	−0.48 (0.15)	−0.32 (0.11)	0.003
*Z*-score (SD)	−3.51 (1.65)	−3.00 (1.35)	0.336	−2.46 (1.25)	−2.28 (0.99)	0.779
% Change (SD)	44.16 (12.75)	−14.34 (7.88)	<0.001	−117.1 (36.3)	−57.0 (15.0)	0.003
SD-index (SD)	−5.22 (1.61)	−1.94 (1.07)	<0.001	−7.50 (2.33)	−5.01 (1.67)	<0.001

Positive response refers to a change of 30% or more in Rrs5 prior to development of airway obstruction (Condition 2). Negative response refers to no change in Rrs5. For Xrs5, a positive response refers to a change of 80% or more. The SD-index was obtained by dividing the change from baseline values by SDw, which was calculated by dividing the difference between the mean values of the 1st and 2nd measurements by √2; Rrs5, resistance at 5 Hz; Xrs5, reactance at 5 Hz; SD, standard deviation; Abs, absolute.
